# CREB-mediated synaptogenesis and neurogenesis is crucial for the role of 5-HT1a receptors in modulating anxiety behaviors

**DOI:** 10.1038/srep29551

**Published:** 2016-07-12

**Authors:** Jing Zhang, Cheng-Yun Cai, Hai-Yin Wu, Li-Juan Zhu, Chun-Xia Luo, Dong-Ya Zhu

**Affiliations:** 1 Department of Pharmacology, School of Pharmacy, Nanjing Medical University, Nanjing 210029, China; 2 Institution of Stem Cells and Neuroregeneration, Nanjing Medical University, Nanjing 210029, China; 3 Institute of Neuroscience, Soochow University, Su zhou, China; 4 The key laboratory of human functional genomics of Jiangsu Province, Nanjing Medical University, Nanjing 210029, China

## Abstract

Serotonin 1a-receptor (5-HT1aR) has been specifically implicated in the pathogenesis of anxiety. However, the mechanism underlying the role of 5-HT1aR in anxiety remains poorly understood. Here we show in mice that the transcription factor cAMP response element binding protein (CREB) in the hippocampus functions as an effector of 5-HT1aR in modulating anxiety-related behaviors. We generated recombinant lentivirus LV-CREB133-GFP expressing a dominant negative CREB which could not be phosphorylated at Ser133 to specifically reduce CREB activity, and LV-VP16-CREB-GFP expressing a constitutively active fusion protein VP16-CREB which could be phosphorylated by itself to specifically enhance CREB activity. LV-CREB133-GFP neutralized 5-HT1aR agonist-induced up-regulation of synapse density, spine density, dendrite complexity, neurogenesis, and the expression of synapsin and spinophilin, two well-characterized synaptic proteins, and abolished the anxiolytic effect of 5-HT1aR agonist; whereas LV-VP16-CREB-GFP rescued the 5-HT1aR antagonist-induced down-regulation of synapse density, spine density, dendrite complexity, neurogenesis and synapsin and spinophilin expression, and reversed the anxiogenic effect of 5-HT1aR antagonist. The deletion of neurogenesis by irradiation or the diminution of synaptogenesis by knockdown of synapsin expression abolished the anxiolytic effects of both CREB and 5-HT1aR activation. These findings suggest that CREB-mediated hippoacampus structural plasticity is crucial for the role of 5-HT1aR in modulating anxiety-related behaviors.

Serotonin (5-HT) contributes to the establishment of anxiety disorders, one of the most prevalent classes of psychiatric disorders^1^. The activity of serotonergic pathways is critically regulated by the plasma membrane serotonin transporter and 5-HT receptors (5-HTRs)^2^. To date, at least 14 different 5-HTR subtypes have been identified in mammals and are grouped into seven families (5-HT_1_-5-HT_7_)^3^. Among them, 5-HT1aR, a metabotropic G protein-coupled receptor widely distributed in the frontal cortex, septum, amygdala, hippocampus, and hypothalamus that receive serotonergic input from the raphe nuclei, plays a major role in modulating the effects of 5-HT on mood and behavior^2^,^4^,^5^. However, the mechanism underlying the role of 5-HT1aR in anxiety remains unknown. Hippocampus is one of several limbic structures involved in the regulation of mood and new-born neurons^6^,^7^, and adult neurognenesis in hippocampus of rodents has been reported to modulate anxiety behavior^8^,^9^. We focused on hippocampus to explore the possible implication of structural plasticity including adult neurogenesis in 5-HT1aR-mediated anxiety modulation.

The cAMP-responsive element-binding protein (CREB), a nuclear transcription factor expressed in all cells in the brain, is best known for its involvement in learning and memory^10^,^11^. That a wide variety of standard antidepressant treatments increase hippocampal CREB activity^10^,^11^,^12^,^13^ implicates CREB in mood disorders. Studies in which CREB knockout mice display increase in anxiety-like behaviors^14^,^15^ and anxiety-like behaviors can be modified by altering CREB function or expression^10^,^16^,^17^,^18^,^19^ suggest a role for CREB in anxiety disorders. Recently, we have reported that 5-HT1aR activation up-regulates phosphorylated CREB (pCREB) level in the hippocampus^20^. In this study, we examined the role of hippocampal CREB in the 5-HT1aR-mediated regulation of anxiety-related behaviors and demonstrated that CREB governs the function of 5-HT1aR by the neuromechanism involving neurogenesis and synaptogenesis.

## Results

### CREB activation is crucial for 5-HT1aR-mediated modulation of anxiety-related behaviors

**Figure 1:**
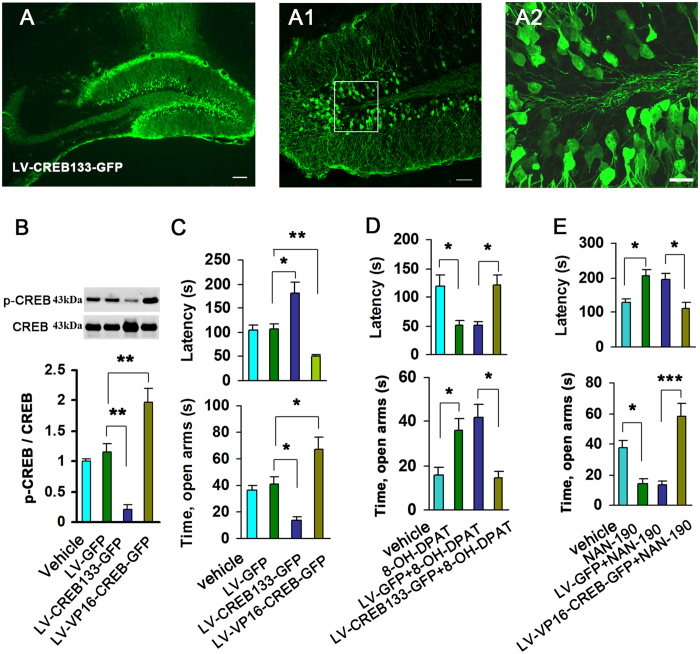
Hippocampal CREB is crucial for the role of 5-HT1aR in modulating anxiety-related bahaviors. (**A**) Representative hippocampal slice infected with LV-CREB133-GFP. Scale bar = 100 μm. (**A1**) A high magnified image of the DG infected with LV-CREB133-GFP. Scale bar = 50 μm. (**A2**) A high magnified image from a selected area in **A1**. Scale bar = 20 μm. (**B**) Immunoblots of pCREB and CREB in the LV-CREB133-GFP- or LV-VP16-CREB-GFP-infected hippocampus (n = 3–4). (**C**) The latency to feed in the NSF test (upper) and time spent in open arms in the EPM test (lower) in the mice whose hippocampal DG was infected by LV-CREB133-GFP or LV-VP16-CREB-GFP or their control (n = 13–15). (**D**,**E**) The latency to feed in the NSF test (upper) and time spent in open arms in the EPM test (lower) in the mice exposed to different treatments (n = 13–15). Means ± SEM. *P < 0.05, **P < 0.01, ***P < 0.001.

To determine the role of hippocampal CREB activity in the modulation of anxiety-related behaviors by 5-HT1aR, we generated recombinant lentivirus LV-CREB133-GFP expressing a dominant negative CREB which could not be phosphorylated at Ser133 to specifically reduce CREB activity, and LV-VP16-CREB-GFP expressing a constitutively active fusion protein VP16-CREB which could be phosphorylated by itself to specifically increase CREB activity^21^. We delivered LV-CREB133-GFP, LV-VP16-CREB-GFP or LV-GFP (2 × 10^8^ infectious units of virus, 2 μl) into bilateral hippocampi of the adult mice by microinjection. Seven days later, we found that they effectively infected the hippocampal dentate gyrus (DG) (Fig. 1A), and LV-CREB133-GFP significantly decreased pCREB level (*F*_(*3,12*)_ = 29.31, *P* = 0.003), whereas LV-VP16-CREB-GFP notably increased pCREB level (*F*_(*3,12*)_ = 29.31; *P* = 0.009) in the hippocampus (Fig. 1B). We performed the novelty suppressed feeding (NSF) and elevated plus maze (EPM) tests at d 21 after infections. In the NSF test, LV-VP16-CREB-GFP caused a significantly decreased latency to feed in novel environment (*F*_(*3,51*)_ = 15.01, *P* = 0.045), while LV-CREB133-GFP produced a significantly increased latency to feed in novel environment (*F*_(*3,51*)_ = 15.01, *P* = 0.007) (Fig. 1C). Neither of groups had effect on the latency to feed in home cage (*F*_(*3,51*)_ = 0.55, P > 0.05) and food consumption in NSF test (*F*_(*3,51*)_ = 1.38, P > 0.05) (data not shown). Similarly, the EPM test revealed that LV-VP16-CREB-GFP significantly increased time spent in open arms (*F*_(*3,51*)_ = 13.95, *P* = 0.023), whereas LV-CREB133-GFP significantly decreased time spent in open arms (*F*_(*3,51*)_ = 13.95, *P* = 0.025), and both of them did not change the total time of entered arms in EPM test (*F*_(*3,51*)_ = 2.09; *P* > 0.05) (data not shown).

To test whether the regulation of anxiety by 5-HT1aR dependents on CREB activation, we treated the LV-CREB133-GFP-infected mice at d 7 after virus infection with 8-OH-DPAT (i.p., 0.1 mg/kg/d × 21 d), a selective 5-HT1aR agonist, and performed the NSF and EPM tests at 24 h after the last 8-OH-DPAT injection. Diminution of CREB phosphorylation by LV-CREB133-GFP abolished the anxiolytic-like effect of 8-OH-DPAT in the NSF (*F*_(*3,59*)_ = 8.23; *P* = 0.01) and EPM tests (*F*_(*3,59*)_ = 7.06; *P* = 0.003) (Fig. 1D). Similarly, we treated the LV-VP16-CREB-GFP-infected mice at d 7 after virus infection with NAN-190 (i.p., 0.3 mg/kg/d × 21d), a selective 5-HT1aR antagonist, and performed the NSF and EPM tests at 24 h after the last NAN-190 injection. Activating CREB reversed the anxiogenic-like effect of NAN-190 in the NSF (*F*_(*3,52*)_ = 21.78; *P* < 0.001) and EPM tests (*F*_(*3,52*)_ = 16.53; *P* < 0.001) (Fig. 1E). These treatments did not alter the latency to feed in home cage and the amount of food consumption in the NSF test and the total number of all entered in arms in EPM test (*P* > 0.05) (data not shown). Together, these data demonstrate that hippocampal CREB is crucial for the role of 5-HT1aR in modulating anxiety-related behaviors.

### CREB activation is responsible for 5-HT1aR-mediated synaptogenesis

**Figure 2:**
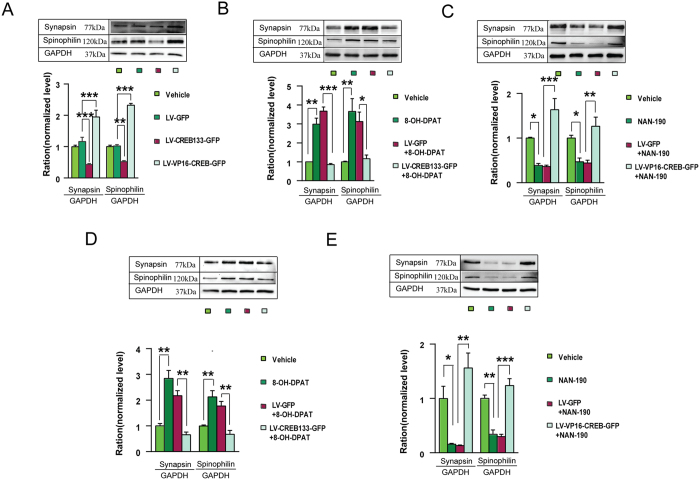
CREB activation is responsible for 5-HT1aR-mediated expression of synapse-related proteins. (**A**) Immunoblots of spinophilin and synapsin in the hippocampus of adult mice infected with different recombinant viruses. (n = 3–4). (**B**,**C**) Immunoblots of spinophilin and synapsin in the hippocampus of adult mice exposed to different treatments. Recombinant viruses were delivered into the DG. (n = 3–5 in (**B)**; n = 3–4 in (**C)**). (**D**,**E**) Immunoblots of spinophilin and synapsin in the cultured hippocampal neurons exposed to different treatments. (n = 3–5 in (**D)**; n = 3–4 in (**E**)). Means ± SEM. *P < 0.05 **P < 0.01, ***P < 0.001.

Stimulation of 5-HT1aR promotes CREB activity^20^,^22^ and synaptogenesis^23^. To test whether 5-HT1aR-mediated synaptogenesis depends on CREB activation, we infused LV-CREB133-GFP or LV-VP16-CREB-GFP into the hippocampal DG of mice by microinjection, and at 21 d after virus infection, we measured expressions of synapsin and spinophilin, two well-characterized synaptic proteins^24^,^25^. As expected, while LV-VP16-CREB-GFP caused significant increases in expressions of synapsin (*F*_(3,24)_ = 23.21, *P* = 0.003) and spinophilin (*F*_(*3,20*)_ = 287.16, *P* < 0.001), LV-CREB133-GFP reduced them substantially (for synapsin, *F*_(*3,24*)_ = 23.21, *P* = 0.06; for spinophilin, *F*_(*3,20*)_ = 287.16, *P* < 0.001) (Fig. 2A).

Next, we treated the LV-CREB133-GFP-infected mice with 8-OH-DPAT (i.p., 0.1 mg/kg/d, × 21 d) and the LV-VP16-CREB-GFP-infected mice with NAN-190 (i.p., 0.3 mg/kg/d, × 21 d) starting at d 7 after virus infection. LV-CREB133-GFP cancelled the effect of 8-OH-DPAT on expressions of synapsin (*F*_(*3,8*)_ = 52.87; *P* < 0.001) and spinophilin (*F*_(*3,16*)_ = 9.83; *P* = 0.041) (Fig. 2B) and LV-VP16-CREB-GFP reversed the effect of NAN-190 on expressions of synapsin (*F*_(*3,24*)_ = 22.17; *P* < 0.001) and spinophilin (*F*_(*3,20*)_ = 11.65; *P* = 0.001) (Fig. 2C).

Moreover, we exposed the LV-CREB133-GFP- or LV-GFP-infected or vehicle-treated hippocampal neurons to 8-OH-DPAT (10^−5^ M) for 48 h (starting at d 8 after virus infection) and found that LV-CREB133-GFP blocked 8-OH-DPAT-induced increases in expressions of spinophilin (*F*_(*3,16*)_ = 15.77, *P* = 0.003) and synapsin (*F*_(*3,8*)_ = 26.69; *P* = 0.005) (Fig. 2D). Similarly, we exposed the LV-VP16-CREB-GFP- or LV-GFP-infected or vehicle-treated hippocampal neurons to 10 μM NAN-190 for 48 h (starting at d 8 after virus infection) and found that LV-VP16-CREB-GFP rescued NAN-190-induced reduction in expressions of synapsin (*F*_(*3,12*)_ = 15.16, *P* = 0.001) and spinophilin (*F*_(*3,16*)_ = 31.64, *P* < 0.001) (Fig. 2E).

**Figure 3:**
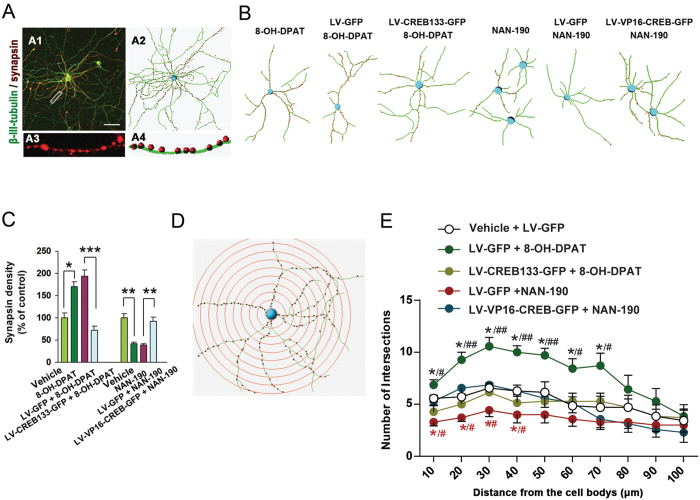
CREB activation is essential for the role of 5-HT1aR in modulating synapsin density and dendrite growth. (**A**) Representative immunofluorescence of synapsin and β-III-tubulin (**A1**) from the cultured hippocampal neurons and simulated diagram view of processes (**A2**). A high magnified image (**A3**) and simulated diagram view (**A4**) from a selected area in **A1**. Scale bar = 50 μm. (**B**) Simulated diagram views of β-III-tubulin and synapsin immunofluorescence from the cultured hippocampal neurons exposed to different treatments. (**C**) Summarized assay of density of synapsin in (**B**). (n = 6). (**D**) A representative simulated diagram view of sholl analysis of dendritic complexity from the hippocampal neurons. (**E**) Sholl analysis of dendritic complexity of the cultured hippocampal neurons exposed to different treatments in (**B**). (n = 7). Means ± SEM. In **C**, *P < 0.05 **P < 0.01, ***P < 0.001; in **E**, *^(*black*)^*P* < 0.05, LV-GFP + 8-OH-DPAT vs LV-GFP + vehicle; *^(*red*)^*P* < 0.05, LV-GFP + NAN-190 vs LV-GFP + vehicle; ^*#*(*black*)^*P* < 0.05, ^*##*(*black*)^*P* < 0.01, LV-CREB133-GFP + 8-OH-DPAT vs LV-GFP + 8-OH-DPAT; ^*#*(*red*)^*P* < 0.05, LV-VP16-CREB-GFP + NAN-190 vs LV-GFP + NAN-190.

To assess synaptogenesis, we co-labelled the neurons with synapsin, a major presyanptic terminals protein, and β-III-tubulin, a major neuron cytoskeleton associated protein, and counted the number of synapsin in fixed-length of process to get the density of synapse (Fig. 3A,B). We exposed the LV-CREB133-GFP- or LV-GFP-infected or vehicle-treated hippocampal neurons to 8-OH-DPAT (10^−5^ M) for 48 h (starting at d 8 after virus infection) and observed synapsin density at 14 d after virus infection. We found that LV-CREB133-GFP blocked 8-OH-DPAT-induced increases in synapse density (*F*_(*3,20*)_ = 18.63, *P* < 0.001). Similarly, we exposed the LV-VP16-CREB-GFP- or LV-GFP-infected or vehicle-treated hippocampal neurons to NAN-190 (10^−5^ M) for 48 h (starting at d 8 after virus infection) and observed synapsin density at 14 d after virus infection. We showed that LV-VP16-CREB-GFP rescued NAN-190-induced decrease in synapse density (*F*_(*3,21*)_ = 14.90, *P* = 0.001) (Fig. 3C). Moreover, we examined the effects of these treatments on dendrite growth (Fig. 3D), and found that LV-CREB133-GFP neutralized 8-OH-DPAT-induced increase in dendritic complexity, whereas LV-VP16-CREB-GFP rescued NAN-190-induced decrease in dendritic complexity (Fig. 3E).

**Figure 4:**
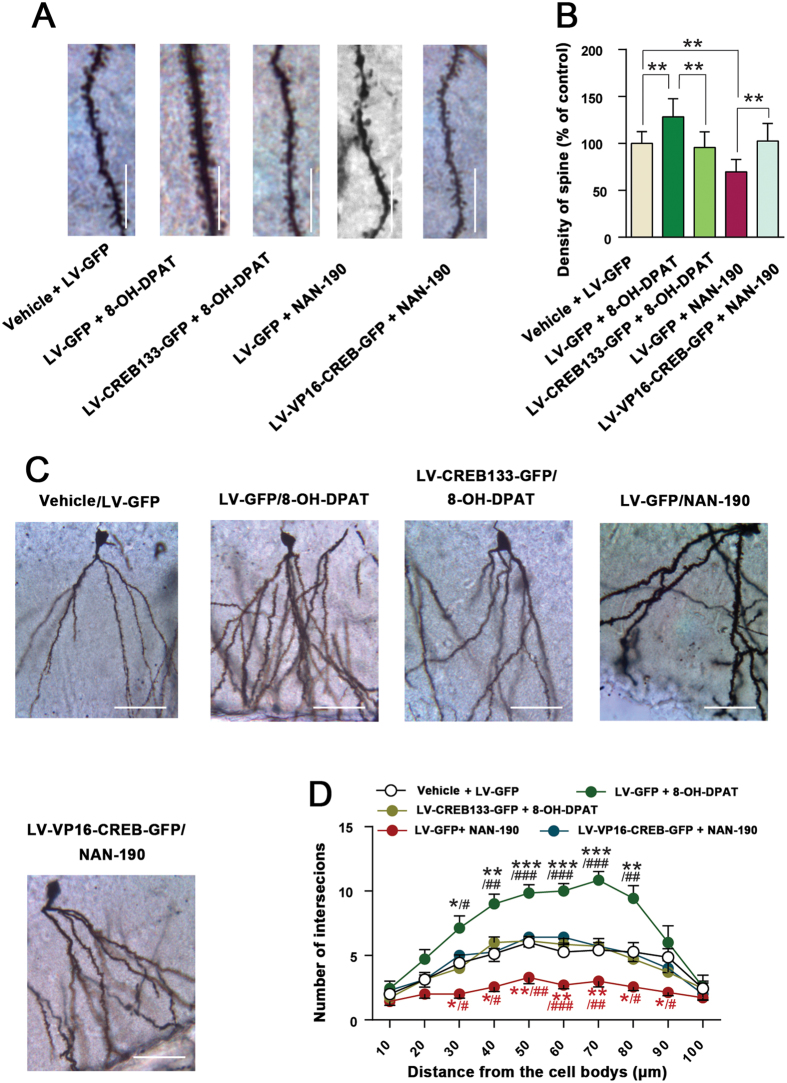
Hippocampal CREB is essential for the role of 5-HT1aR in modulating synaptogenesis. (**A**) Representative images with Golgi-Cox staining showing dendrite spine density of granular cells in the DG of mice treated by different treatments. Scale bar = 40 μm. (B) Bar graph showing dendritic spine density in (**A**) (n = 8). (**C**) Representative images with Golgi-Cox staining showing granular cells in the DG of mice exposed to treatments as in (**A**). Scale bar = 100 μm. (**D**) Sholl analysis of dendritic complexity of Golgi-stained neurons in (**C**). (n = 8). Means ± SEM. In **B**, *P < 0.05, **P < 0.01, ***P < 0.001. In **D**, *^(*black*)^*P* < 0.05, **^(*black*)^*P* < 0.01, ***^(*black*)^*P* < 0.001; LV-GFP + 8-OH-DPAT vs LV-GFP + vehicle; *^(*red*)^*P* < 0.05, **^(*red*)^*P* < 0.01, LV-GFP + NAN-190 vs LV-GFP + vehicle; ^*#*(*black*)^*P* < 0.05, ^*##*(*black*)^*P* < 0.01, ^*##*(*black*)^*P* < 0.001, LV-CREB133-GFP + 8-OH-DPAT vs LV-GFP + 8-OH-DPAT; ^*#*(*red*)^*P* < 0.05, ^*##*(*red*)^*P* < 0.01, ^*###*(*red*)^*P* < 0.001, LV-VP16-CREB-GFP + NAN-190 vs LV-GFP + NAN-190.

To further strengthen our findings, we infused LV-CREB133-GFP or LV-GFP into the hippocampal DG of mice by microinjection, and 7 days later, delivered 8-OH-DPAT (10^−5^ M, 2 μl) into the hippocampus, and measured dendritic branching and the number of dendritic spines by Golgi staining 14 d after virus infection. Activation of 5-HT1aR by 8-OH-DPAT significantly increased spine density (*F*_(*4,35*)_ = 36.80; P = 0.001) and dendritic complexity, and LV-CREB133-GFP neutralized the effect of 8-OH-DPAT on spine density (*F*_(*4,35*)_ = 36.80; P < 0.001) and dendritic complexity (Fig. 4A–D). Similarly, we infused LV-VP16-CREB-GFP or LV-GFP into the hippocampus of mice by microinjection, and 7 days later, delivered NAN-190 (10^−5^ M, 2 μl) into the hippocampus, and measured dendritic branching and the number of dendritic spines by Golgi staining 14 d after virus infection. Blockade of 5-HT1aR by NAN-190 significantly decreased spine density (*F*_(*4,35*)_ = 36.80; P < 0.001) and dendritic complexity, and LV-VP16-CREB-GFP rescued the effect of NAN-190 on spine density (*F*_(*4,35*)_ = 36.80; P < 0.001) and dendritic complexity (Fig. 4A–D). Taken together, our findings suggest that CREB activation is crucial for 5-HT1aR-mediate synaptogenesis.

### CREB activation accounts for the neurogenic role of 5-HT1aR

**Figure 5:**
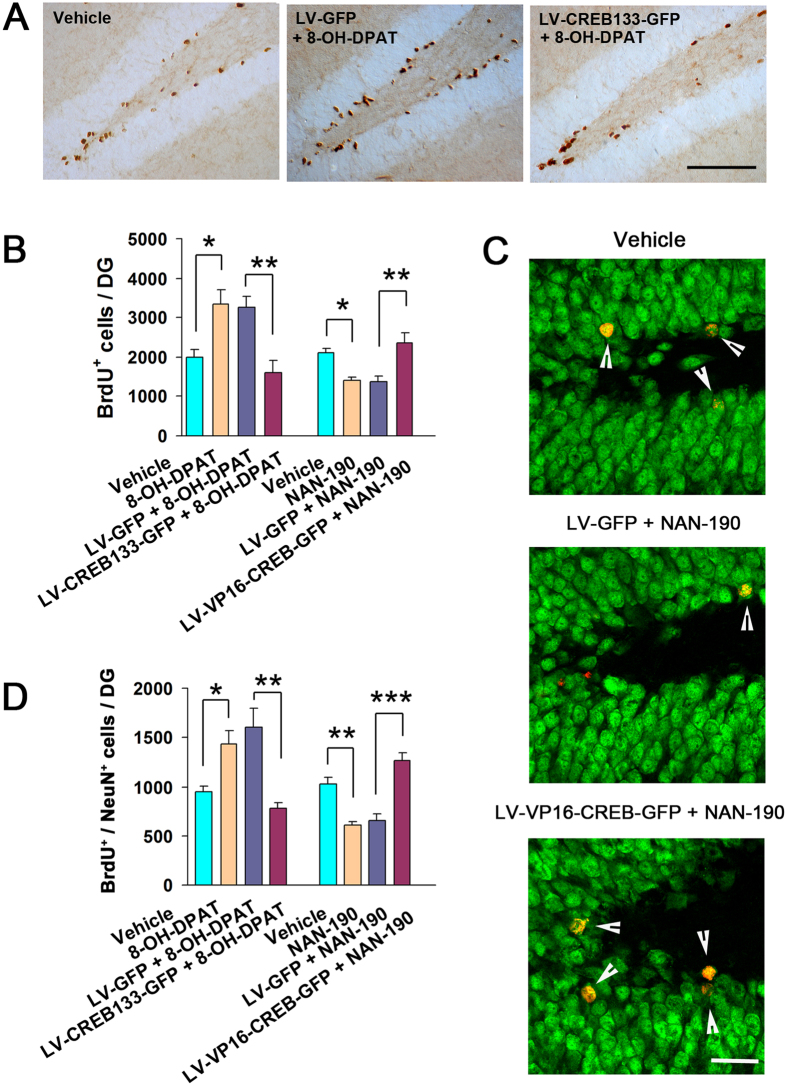
CREB activation accounts for the neurogenic role of 5-HT1aR. (**A**) Representatives showing BrdU^+^ cells in the DG of mice exposed to different treatments at 12 h after BrdU labeling. (**B**) Statistical graphs showing the number of BrdU^+^ cells in the DG of mice exposed to different treatments at 12 h after BrdU labeling (n = 5). Scale bar = 200 μm. (**C**) Representative images of BrdU^+^/NeuN^+^ cells in the DG of mice exposed to different treatments at 28 d after BrdU labeling. Scale bar, 50 μm. (**D**) Statistical graph showing the number of BrdU^+^/NeuN^+^ in the DG of mice exposed to different treatments at 21 d after BrdU labeling (n = 5). Scale bar = 50 μm. Means ± SEM. *P < 0.05,**P < 0.01, ***P < 0.001.

Hippocampal neurogenesis is required for anxiolytic effects of chronic antidepressants^26^ and stimulation of 5-HT1aR up-regulates the adult hippocampal neurogenesis^27^. Accordingly, we investigated whether CREB activation explains the neurogenic role of 5-HT1aR. We delivered LV-CREB133-GFP, LV-VP16-CREB-GFP, LV-GFP or vehicle into bilateral hippocampi of the adult mice, and 7 days later, treated these mice with 8-OH-DPAT (i.p., 0.1 mg/kg/d × 21 d) or NAN-190 (i.p., 0.3 mg/kg/d × 21 d). At 24 h after the last 8-OH-DPAT or NAN-190 injection, these mice were injected with BrdU (50 mg/kg, i.p., for 4 times at 12h intervals) and sacrificed at 12 h after the last BrdU injection. Interestingly, LV-CREB133-GFP abolished 8-OH-DPAT-induced enhancement (*F*_(*3,15*)_ = 11.02, *P* = 0.031) and LV-VP16-CREB-GFP reversed NAN-190-iduced reduction (*F*_(*3,15*)_ = 11.02, *P* = 0.005) in proliferation (Fig. 5A,B).

To test the role of CREB activation in 5-HT1aR-mediated neuronal differentiation of newborn cells, we delivered LV-CREB133-GFP, LV-VP16-CREB-GFP, LV-GFP or vehicle into bilateral hippocampi of the adult mice, and 7 days later, treated these mice with 8-OH-DPAT (i.p., 0.1 mg/kg/d × 21 d) or NAN-190 (i.p., 0.3 mg/kg/d × 21 d). At 24 h after the last 8-OH-DPAT or NAN-190 injection, these mice were injected with BrdU (50 mg/kg, i.p., for 4 times at 12h intervals) and sacrificed at 28 d after the last BrdU injection. As shown in Fig. 5C,D, 8-OH-DPAT significantly increased the number of BrdU^+^/NeuN^+^ cells in the DG (*F*_(*3,16*)_ = 12.86, *P* = 0.048) and the effect of 8-OH-DPAT was abolished by LV-CREB133-GFP (*F*_(*3,16*)_ = 12.86, *P* = 0.001). Contrastingly, NAN-190 significantly decreased the number of BrdU^+^/NeuN^+^ cells in the DG (*F*_(*3,16*)_ = 22.96, *P* = 0.001) and LV-VP16-CREB-GFP rescued the NAN-190-induced decrease in neurons production (*F*_(*3,16*)_ = 22.96, *P* < 0.001). Thus, CREB phosphorylation plays a role in 5-HT1aR-mediated neurogenesis and newborn neurons.

### Regulation of anxiety-related behaviors by 5-HT1aR-CREB signaling depends on neurogenesis and synaptogenesis

**Figure 6:**
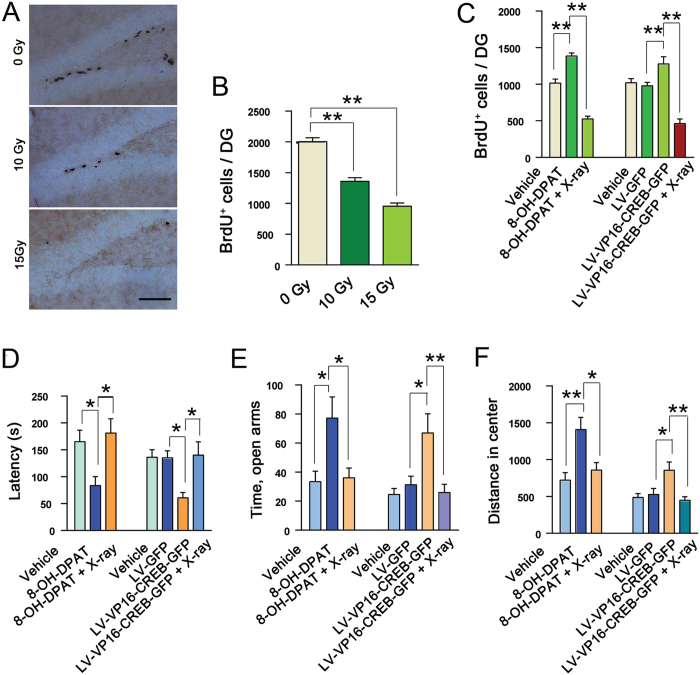
Neurogenesis is required for the regulation of anxiety-related behaviors by 5-HT1aR-CREB signaling. (**A**) Representatives showing BrdU^+^ cells in the DG of mice exposed to different doses of X-ray. Scale bar = 200 μm. (**B**) Total number of BrdU^+^ cells per DG from mice exposed to different doses of X-ray (n = 6). (**C**) Total number of BrdU^+^ cells per DG from mice exposed to different treatments (n = 6). (**D**–**F**) X-ray abolished behavioral effects of 8-OH-DPAT or VP16-CREB in the latency to feed in the NSF test (n =  15, **D**), time spent in open arms in the EPM test (n = 15, **E**) and the distance in center of OF test (n = 14–15, **F**). Means ± SEM. *P < 0.05,**P < 0.01, ***P < 0.001.

To determine whether neurogenesis is required for the role of 5-HT1aR-CREB signaling in modulating anxiety behaviors, we sought to disrupt this process by X-irradiation^28^. We delivered 2 doses of 5 Gy X ray (at d 1 and 4 respectively) or 3 doses of 5 Gy X ray (at d 1, 4 and 8 respectively) (total 10 or 15 Gy) to the hippocampus of mice, injected BrdU (200 mg/kg, i.p.) to label newly born cells in the hippocampus at 2 h after last irradiation and performed BrdU staining at 12 h after BrdU labeling. As shown in Fig. 6A,B, 10Gy X ray resulted in 32.2% reduction and 15 Gy X ray resulted in 52.4% reduction in BrdU^+^ cells.

Next, we infused 8-OH-DPAT (10^−5^ M, 2 μl) into the bilateral DG of mice, delivered 15 Gy X ray to the hippocampus beginning from d 4 after 8-OH-DPAT microinjection, and injected BrdU (200 mg/kg, i.p.) to label newly born cells in the hippocampus at 2 h after last irradiation. We performed behavioral tests at d 28 after the last dose of X ray and BrdU staining at d 30 after BrdU labeling. Treatment with 8-OH-DPAT caused 30.5% increase in the survival of newly born cells and X-irradiation abolished the effects of 8-OH-DPAT on survival (*F*_(2,15)_ = 109.96, *P* < 0.001) (Fig. 6C) and on the latency to feed in novel enviroment in the NSF test (*F*_(2,42)_ = 5.70, *P* = 0.012) (Fig. 6D), time spent in open arms in EPM test (*F*_(*2,42*)_ = 5.84, *P* = 0.024) (Fig. 6E) and distance in center in OF test (*F*_(*2,42*)_ = 8.24, *P* = 0.014) (Fig. 6F). The latency to feed in home cage and the amount of food consumption in NSF test, the total number of all entered fields in EPM test, and basic movements in OF test in all groups were similar (*P* > 0.05, data not shown).

To further ascertain this finding, we infused LV-VP16-CREB-GFP into the bilateral DG of mice by microinjection and delivered 15 Gy X ray to the hippocampus beginning from d 8 after virus infection and injected BrdU (200 mg/kg, i.p.) to label newly born cells in the hippocampus at 2 h after last irradiation. We performed behavioral tests at d 28 after the last dose of X ray and BrdU staining at d 30 after BrdU labeling. LV-VP16-CREB-GFP caused 40.2% increase in the survival of newly born cells and X-irradiation abolished the effects of LV-VP16-CREB-GFP on survival (*F*_(*3,20*)_ = 60.48, *P* < 0.001) (Fig. 6C), the latency to feed in novel environment in NSF test (*F*_(*3,55*)_ = 5.18, *P* = 0.015) (Fig. 6D), time in open arms in EPM test (*F*_(*3,55*)_ = 6.35, *P* = 0.007) (Fig. 6E) and distance in center in OF test (*F*_(*3,55*)_ = 5.73, *P* = 0.006) (Fig. 6F). The latency to feed in home cage and the amount of food consumption in NSF test, the total number of all entered fields in EPM test, and basic movements in OF test in all groups were similar (*P* > 0.05, data not shown).

**Figure 7:**
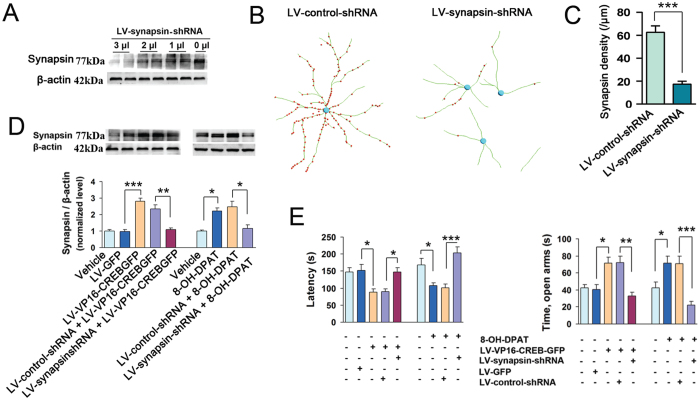
Regulation of anxiety-related behaviors by 5-HT1aR-CREB signaling depends on synaptogenesis. (**A**) Simulated diagram views of synapsin immunofluorescence from the cultured hippocampal neurons infected by LV-synapsin-shRNA or LV-control-shRNA. (**B**) Immunoblot of synapsin in the hippocampus of adult mice exposed to LV-synapsin-shRNA at the doses indicated (n = 3). (**C**) Summarized assay of synapsin density in the cultured hippocampal neurons incubated with LV-synapsin-shRNA or its control for 10 d. (n = 14). (**D**) Immunoblot of synapsin in the hippocampus of adult mice exposed to different treatments (n = 4–5). (**E**) The latency to feed in the NSF test (left) and time spent in open arms in the EPM test (right) in the mice exposed to different treatments (n = 14–15). Means ± SEM. *P < 0.05, **P < 0.01, ***P < 0.001.

To determine whether synaptogenesis accounts for the role 5-HT1aR-CREB signaling in modulating anxiety behaviors, we attempted to disrupt this process by shRNA^29^. We generated recombinant lentivirus LV-synapsin-shRNA expressing the shRNA of synapsin. We infused 1, 2 or 3 μl of LV-synapsin-shRNA into the hippocampus by microinjection to knockdown synapsin, and 7 days later, we measured the expression of synapsin in hippocampus. As shown in Fig. 7A, LV-synapsin-shRNA inhibit the expression of synapsin dose-dependently (*F*_(*3,20*)_ = 16.8, *P* < 0.001). At the dose of 2 μl, LV-synapsin-shRNA significantly decreased synapsin density of hippocampal neurons (*F*_(*1,26*)_ = 53.15, *P* < 0.001) (Fig. 7B,C).

Next, we test whether LV-synapsin-shRNA abolishes LV-VP16-CREB-GFP- or 8-OH-DPAT-induced synapsin expression and behavioral modification. We delivered LV-synapsin-shRNA (2 μl) into the bilateral DG of mice by microinjection, and 7 days later, we infused LV-VP16-CREB-GFP or 8-OH-DPAT (10^−5^ M, 2 μl) into the DG and measured the expression of synapsin in the hippocampus at d 21, and performed behavioral tests at d 28 after LV-VP16-CREB-GFP infection or 8-OH-DPAT microinjection. LV-VP16-CREB-GFP significantly increased synapsin expression (*F*_(4,20)_ = 28.20, *P* < 0.001), and LV-synapsin-shRNA abolished the effects of LV-VP16-CREB-GFP on synapsin expression (*F*_(4,20)_ = 28.20; *P* = 0.001) (Fig. 7D), on the latency to feed in novel enviormnet in NSF test (*F*_(4,76)_ = 6.92, *P* = 0.038) and time in open arms in EPM test (*F*_(4,76)_ = 9.98, *P* = 0.001) (Fig. 7E). Similarly, 8-OH-DPAT significantly increased synapsin expression (*F*_(3,16)_ = 9.96, *P* = 0.018) (Fig. 7D), and LV-synapsin-shRNA abolished the effects of 8-OH-DPAT on synapsin expression (*F*_(3,16)_ = 9.96, *P* = 0.011) (Fig. 7D), on the latency to feed in novel enviormnet in NSF test (*F*_(3,63)_ = 11.45, *P* < 0.001) and time in open arms in EPM test (*F*_(3,63)_ = 11.80, *P* < 0.001) (Fig. 7E). The latency to feed in home cage and the amount of food consumption in NSF test, the total number of all entered fields in EPM test, and basic movements in OF test in all groups were similar (*P* > 0.05, data not shown). Collectively, knockdown of synapsin abolishes anxiolytic-like effects produced by 5-HT1aR activation or CREB phosphorylation, which suggest that synaptogenesis is critical for 5-HT1aR-CREB signaling-mediated modification of anxiety behaviors.

## Discussion

Mechanisms that underlie anxiety remain poorly understood. Dozens of reports indicate that 5-HT1aR and CREB activation are implicated in the modulation of anxiety-related behaviors. Here, we report that 5-HT1aR, acting via activating CREB in the hippocampus, promotes neurogenesis and synaptogenesis, thereby modulates anxiety-related behaviors.

The 5-HT1aR is a metabotropic G protein-coupled receptor and has been specifically implicated in the pathogenesis of depression and anxiety^30^. Activation of 5-HT1aR is critical for the anxiolytic effects of SSRIs^26^, the first-line compounds for clinical treatment of anxiety-related disorders^30^. Knockout of 5-HT1aR leads to an anxiogenic phenotype^26^ and tissue-specific expression of 5-HT1aR in the hippocampus and cortex of 5-HT1aR KO mice recovers wild-type phenotype^31^. Moreover, 5-HT1aR agonists have been clinically used as anxiolytic drugs^32^. CREB knockout mice exhibit increase in anxiety-like behaviors^15^ and anxiety-like behaviors can be modified by altering CREB function or expression^10^,^16^,^17^,^18^,^19^, and chronic antidepressants administration increases CREB expression, phosphorylation and function in limbic brain structures, including hippocampus and cerebral cortex^12^, suggesting a role for CREB in anxiety disorders. We recently demonstrated that 5-HT1aR mediates pCREB up-regulation in the hippocampus^20^. By using LV-CREB133-GFP expressing a dominant negative CREB, and LV-VP16-CREB-GFP expressing a constitutively active fusion protein VP16-CREB, present study indicates that CREB in the hippocampus functions as an effector of 5-HT1aR in modulating anxiety-related behaviors. Disrupting CREB activity abolished the anxiolytic effect of 5-HT1aR agonist and elevating CREB activity reversed the anxiogenic effect of 5-HT1aR antagonist. Hippocampus structural plasticity may explain the role of 5-HT1aR-CREB signaling in modulating anxiety-relative behaviors. Hippocampus is one of several limbic structures that have been associated with anxiety disorders^6^,^7^. Adult neurogenesis in the hippocampal DG represents a unique form of hippocampal structural and functional plasticity^33^. Studies have confirmed that hippocampal neurogenesis is necessary for the anxiolytic effects of antidepressants^22^,^26^. The 5-HT1aR subtype is predominantly postsynaptic to 5-HT neurons in the hippocampus^34^. The hippocampal postsynaptic 5-HT1aR is crucial for modulating anxiety-related behavior and neurogenesis^26^,^27^,^31^. CREB-dependent signaling controls essential developmental steps in adult neurogenesis, including survival, maturation and integration of new neurons^35^. We showed that CREB activation is responsible for 5-HT1aR-mediated neurogenesis. More importantly, disrupting hippocmapal neurogenesis by irradiation abolished the anxiolytic effects of both CREB and 5-HT1aR activation, suggesting the requirement of neurogenesis for the regulation of anxiety-related behaviors by 5-HT1aR-CREB signaling. Although new neurons produced in the adult brain represent only a relatively small proportion of the total population of mature granule neurons, the unique properties of adult-generated neurons that are structurally plastic during their immature stages^36^ and with different electrophysiological characteristics from those of mature neurons^37^,^38^,^39^ may answer why so few neurons have functional significance. However, we can not exclude the effect of irradiation alone on behaviors, as some researchers found irradiation alone may influence hippocampal 5-HT1aR function^40^.

Activity-induced changes in synaptic structure are related to anxiety-related behaviors^41^. A notable recent discovery shows that ketamine, a NMDAR antagonist, rapidly reverses chronic stress-induced anxiety by inducing synaptogenesis^42^. Stimulation of the hippocampal 5-HT1aR caused a dramatic increase in PSD95 expression and dendritic spine and synapse formation through sequential activation of the mitogen-activated protein kinase isozymes Erk1/2 and protein kinase C^23^. CREB activation has opposite effects on synaptogenesis in different contexts. For mature neurons, several studies have shown a correlation between increased CREB activity and enhanced synaptogenesis. In contrast, CREB activation suppresses synaptogenesis and reduces network synchrony in early development^43^. In the present study, our results suggest that 5-HT1aR-mediated synaptogenesis depends on CREB activity. Similar to the disruption of neurogenesis, blocking synaptogenesis by the knockdown of synapsin expression in the hippocampus abolished the anxiolytic effects of both CREB and 5-HT1aR activation.

Though structural plasticity is demonstrated crucial for the role of 5-HT1a receptors in modulating anxiety behaviors by our study, we can not exclude other possibilities. For example, increased oxidative stress^44^ and over-activation of hypothalamic-pituitary-adrenal (HPA) axis^45^ were also supposed as mechanisms underlying anxiety behaviors. Whether the effect of 5-HT1a receptors in anxiety modulation is associated with oxidative stress or HPA axis is remained unknown.

In sum, our data highlight the important significance of CREB activation in the hippocampus for the 5-HT1aR-mediated modulation of anxiety-related behaviors. Stimulation of 5-HT1aR up-regulates CREB phosphorylation, and in turn, promotes neurogenesis and synaptogenesis, thereby leads to anxiolytic-like behaviors. However, we could not distinguish the role of CREB between the proliferating precursor cells and the mature granule cells, as the whole dentate gyrus of hippocampus was treated without a cell-specific approach in this study. Although CREB activity in both cells contributes to 5-HT1aR-mediated modulation of anxiety-related behaviors, it is likely that CREB in precursor cells and in mature cells is responsible for neurogenesis and synaptogensis, respectively, according to our results.

## Materials and Methods

### Animals

Young adult (6- to 7-week-old) male ICR mice, and newborn [postnatal day P0–P1] ICR mice (from Model Animal Research Center of Nanjing University, Nanjing, China) were used in this study. The outbred ICR mice shows the more active exploratory behavior and the faster pace of adaptation to the new environment than the outbred BALB/c and C57BL/6 mice^46^ and is sensitive to anxiety behavior tests^20^. The mice were maintained at a controlled temperature (20 ± 2 °C) and group housed (12 h light/dark cycle) with access to food and water ad libitum. All procedures involving the use of animals were approved by the Institutional Animal Care and Use Committee of Nanjing Medical University. And all experiments were performed in accordance with the approved guidelines and regulations. Every effort was made to minimize the number of animals used and their suffering.

### Drugs

8-OH-DPAT, NAN-190 and BrdU were purchased from Sigma-Aldrich.

### Intrahippocampal microinjections

Adult mice were anesthetized with chloral hydrate (400 mg/kg, i.p.), and placed in a stereotaxic apparatus. For the microinjections, 8-OH-DPAT (3.28 μg/1.0 μl) or NAN-190 (4.74 μg/1.0 μl) solution in 1.0 μl volume was positioned at the following coordinates: 2.3 mm posterior to bregma, 1.3 mm lateral to the midline, and 2.0 mm below dura^20^ and injected into the hippocampus (0.2 μl/min).

### Behaviors tests

The anxiety-like behaviors in mice were assessed with three widely used conflict paradigms: novelty suppressed feeding (NSF), elevated plus maze (EPM) and open field (OF). EPM and OF tests quantify anxieties elicited by natural aversive stimuli, and NSF test quantify anxieties elicited by acute stressors^47^.

The NSF is a conflict test that elicits competing motivations: the drive to eat and the fear of venturing into the center of brightly lit arena^48^, which depends less on locomotor activity^49^. The latency to feed in NSF test has been used as an index of anxiety-like behaviors^26^. The NSF test was carried out during a 5-min period as described previously^50^. In brief, the testing apparatus consisted of a plastic box (50 × 50 × 20 cm), the floor of which was covered with approximately 2 cm of wooden bedding. Twenty-four hours before behavioral testing, all the food was removed from the home cage. At the time of testing, a single pellet of food (regular chow) was placed on a white paper platform positioned in the center of the box. Each mouse was placed in a corner of the box, facing the corner and a stopwatch was immediately started. The latency to eat (defined as the mouse sitting on its haunches and biting the pellet with the use of forepaws) was timed. Immediately after this test, the animal was transferred to its home cage, and the latency to eat and the amount of food consumed by the mouse in 15 min was measured, serving as a control for change in appetite as a possible confounding factor.

The EPM also presents a conflict between two “risk-laden” arms (open without sidewalls, 30 × 5 cm) and two “safe” arms (closed by sidewalls, 30 × 5 cm, with end and side walls 15 cm high), connected with a central platform (5 × 5 cm). The EPM tests were assessed at 24 h after the NSF test. The maze was raised to a height 38.5 cm above the floor. Each mouse was placed in the intersection of the four arms of the maze, facing an open arm and allowed to explore freely for 5 min. An arm entry was defined as a mouse entering an arm of the maze with all four legs. General activity was evaluated using the total number of entries into the arms. Anxiety was assessed using time spent in open arms.

The OF is an arena that presents a conflict between innate drives to explore a novel environment and safety. Under brightly lit conditions, the center of the OF is aversive and potentially risk-laden, whereas exploration of the periphery provides a safer choice. In mice it has been shown that anxiety animals present more exploration of the periphery in OF test^49^. OF activity assay was performed at 24 h after the NSF test using the MotorMonitor System SF16R (CA, USA) on PC computer. The test arena was constructed of a plastic plate (56.13 × 56.13 cm) and divided into 256 squares by lines drawn on the floor of the plate. It was surrounded by a 35.18 cm high plastic wall. Each mouse was placed onto a corner square of the arena, facing the corner and allowed to freely explore the open field for 5 min per trial. During the period, the numbers of entered outer and inner squares were counted. An entry into a square was defined as having the two forelimbs in the square at one time. After each trial the plate was cleaned with 70% EtOH.

### Culture of hippocampal neurons

Hippocampi of embryo day 15 (E15) ICR mice were removed and placed in Hanks’ balanced salt solution (HBSS) without Ca^2+^ and mg^2+^ (Gibco BRL, Grand Island, NY) containing 1 mM sodium pyruvate and 10 mM HEPES. Then the hippocampal tissues were dissociated in HBSS solution containing 0.125% trypsin solution for 10 min at 37 °C. Subsequently, tissues were triturated by repeated passage through a constricted Pasteur pipette. The digestion was stopped with DMEM along with 10% heat-inactivated fetal bovine serum. The dispersed tissues were allowed to settle for 3 min. The supernatant was transferred to a fresh tube and centrifuged at 2000 rpm for 2 min. The pellet was resuspended in a neuron-defined culture medium, serum-free neurobasal medium (Gibco), supplemented with B-27, 0.5 mM L-glutamine, 20 IU/ml penicillin and 20 IU/ml streptomycin. The cells were then plated onto 6-well plates coated with poly-D-lysine (100 μg/ml) at 2 × 10^4^/cm^2^. Cell cultures were kept in a humidified atmosphere of 95% air and 5% CO_2_ at 37 °C. Half of the medium was replaced with fresh medium without glutamate every 2–3 days. The purity of neuronal cultures was determined by immunofluorescence using staining with antibody against β-III-tubulin (1:200; Chemicon International Inc.), and the nuclei were stained for 15 min with Hoechst 33258 (1 μg/ml in PBS). The purity of the neurons used to experiments was ~95%.

### Lentivirus Production and Infection

A recombinant lentivirus, LV-CREB133-GFP, was generated with the plasmid pCMV-CREB133 (Clontech Laboratories). LV-CREB133-GFP expresses a mutant variant of the human CREB protein (CREB133) that contains a serine to alanine mutation corresponding to amino acid 133 in the mutant mouse CREB protein. This mutation blocks phosphorylation of CREB, thus preventing transcription. Another recombinant lentivirus, LV-VP16-CREB-GFP, was generated with the plasmid pSK-VP16-CREB (Angel Barco, UMH-CSIC, Spinish). LV-VP16-CREB-GFP expresses the chimeric protein, VP16-CREB, was obtained by replacing the first transactivation domain of CREB with the acidic transactivation domain of Herpes simplex virus (HSV) VP16. Equivalent chimeric proteins bind to CRE sequences in tissue-specific promoters and behave like CREB activated by phosphorylation, thus promoting transcription. The titer of LV-CREB133-GFP and LV-VP16-CREB-GFP was 2 × 10^8^ infectious units of virus (IFU). LV-CREB133-GFP, LV-VP16-CREB-GFP or LV-GFP (control LV) was added into cultured neurons as the amount of neuron (MOI (multiplicity of infection) = 2, MOI = the virus titer/the amount of neuron) when cultured 4 d. Twenty-four hours later, the medium was fully changed with fresh medium without lentrivirus. After infected with the recombinant lentivirus for 5–10 d, neurons were used for experiment. *In vivo*, we infused LV-CREB133-GFP (2 μl) or LV-VP16-CREB-GFP (2 μl) into mice using the intrahippocampal microinjections methods. After infected with the recombinant lentivirus for 7 d, it can stablely express in hippocampus. The procedures concerning recombinant lentivirus were performed following National Institutes of Health guidelines.

### Immunofluorescence

Fixed hippocampal neurons were incubated with rabbit anti-synapsin antibody (1:200; Chemicon International Inc.) and mouse anti-β-III-tubulin (1:200; Chemicon International Inc.), binding was visualized with anti-rabbit Cy3-conjugated secondary antibody (1:200, Jackson) and anti-mouse Dylight-conjugated secondary antibody (1:200, Jackson). Images of immunostained neurons in all groups were captured with a Zeiss Axio Cam MRC 5(D) camera mounted on Carl-Zeiss Axio Observer A1 microscope under same conditions. The Density of synapsin was analyzed using Imairs 7.3.0 (Bitplane, Swiss). We randomly chose 10 synapsin-positive neurons in this area to measure the density of synapsin. The density from sampled neuron was averaged.

### BrdU Immunohistochemisty

Animals were perfused transcardially with 200 ml of 0.05 M sodium phosphate (pH 7.4) containing 0.8% NaCl, followed by 300 ml of 4% paraformaldehyde in 0.05 M sodium phosphate (pH 7.4, containing 0.8% NaCl). BrdU staining was performed as described previously^51^. Brains were removed and post-fixed overnight in the same solution. Serial hippocampal sections (40 μm) were made on an oscillating tissue slicer in a bath of physiological saline. The sections were heated (85 °C for 5 min) in antigen unmasking solution (Vector Laboratories, Burlingame, CA, USA), incubated in 2 M HCl (37 °C for 30 min), rinsed in 0.1 M boric acid, pH 8.5, for 10 min, incubated in 1%H_2_O_2_ in PBS for 30 min and blocked in PBS containing 3% normal goat serum, 0.3% (w/v) Triton X-100 and 0.1% BSA (room temperature for 1 h), followed by incubation with mouse monoclonal anti-BrdU (1:200; Accurate Chemical & Scientific Corporation, Westbury, NY, USA), rabbit monoclonal anti-NeuN (1:400, abcam) at 4 °C overnight. Subsequently, the sections were developed with the ABC kit (Vector Laboratories) or Cy3 fluorescence secondary antibody (1:200, Jackson) and Dylight secondary antibody (1:200, Jackson).

### Golgi-Cox staining

The fresh brain without perfusion were used for Golgi-Cox staining with FD Rapid GolgiStain Kit (FD Neuro Technologies, Columbia, MD,USA) according to the user manual. Briefly, the brain was first placed in impregnation solution for 2 weeks followed by 2 d in 30% sucrose. Then they were cut into 100-μm coronal sections using a vibratome (World Precision Instruments, Sarasota, FL, USA) and stained. The total number of dendritic branches was counted at each order away from cell body or dendritic shaft. To calculate spine density of Golgi-stained neurons in the DG, a length of dendritic was traced, the exact length of the dendritic segment was calculated and the number of spines along that length was counted. The neurons randomly for each sample were measured, and the average was regarded as the final value of one sample.

### Sholl analysis

Images of β-III-tubulin or Golgi-stained neurons were captured with a Zesis Axio microscope using a 40× objective. Each neuron was reconstructed using Imaris 7.5.2 software, and all analyses were performed using the Image J Sholl Analysis Plugin; the center of all concentric circles was defined as the center of cell soma. The starting radius was 10 μm for neurons *in vivo* and *in vitro*, and the ending radius was 100 μm for neurons *in vivo* and *in vitro* from center; the interval between consecutive radii was 10 μm for neurons *in vivo* and *in vitro*.

### Cell counting

One experimenter coded all slides from the experiments before quantitative analysis. Proliferating or surviving cells, detected by their BrdU-positive nuclei, were counted by another experimenter who was unaware of the experimental conditions of each sample. The analysis was conducted on every sixth section in a series of 40 μm coronal sections. To determine the total number of BrdU^+^ cells per SVZ or DG, the counts from sampled sections were averaged and the mean values were multiplied by the total number of sections.

### Western blot analysis

Samples from cultured neurons and hippocampal tissues of animals were prepared as described by our previous studies^52^,^53^. The samples containing equivalent amounts of protein (20 μg) were applied to 8 ~ 12% (8% for synapsin and spinophilin; 12% for CREB, p-CREB and GAPDH) acrylamide denaturing gels (SDS-PAGE). The separated proteins were transferred onto nitrocellulose membranes overnight at 4 °C. Blotting membranes were incubated with blocking solution (5% nonfat dried milk powder dissolved in TBST buffer (pH 7.5, 10 mM Tris-HCl, 150 mM NaCl, and 0.1% Tween 20)) for 1 hour at room temperature, washed three times, and then were incubated with rabbit antiphospho-CREB-ser133 (1:500; Chemicon), or rabbit anti-CREB (1:1000; Chemicon), or mouse anti-synapsin (1:1000; Chemicon), or mouse anti-spinohphilin (1:1000; Chemicon) in TBST overnight at 4 °C. Internal control was carried out using mouse anti-GAPDH (1:2000, Chemicon). After several washes with TBST buffer, the membranes were incubated for 1 h with horseradish peroxidase-linked secondary antibody (1:10000, Liankebio). The membranes were then processed with enhanced chemiluminescence western blotting detection reagents (Pierce). The films were scanned and densitometry was performed using the ‘Quantity One’ image software (Bio-Rad). The relative level of the protein was quantified from the scanned films.

### Hippocampal X-irradiation

X-irradiation was performed according to a modified version of a procedure reported previously^25^. Briefly, mice were anesthetized with chloral hydrate (400 mg/kg, i.p.), placed in a stereotaxic apparatus, and exposed to cranial irradiation using therapeutical x-ray equipment operated at 300 kVp and 20 mA. Mice were protected with a lead shield that covered the entire body, with the exception of a 3.2 × 11 mm treatment field above the hippocampus. The corrected dose rate was 1.2Gy/min at a source-toskin distance of 100 cm. The procedure lasted 4 min and 10 s, delivering a total of 5 Gy each time. For the 15 Gy dose group, three 5 Gy doses were delivered on days 0, 4, and 8, respectively. To assess the effects of this procedure on the survival of newly born cells, mice were given injections of BrdU (four times, 50 mg/kg, i.p., at 6 h intervals) on day 12. Behavioral tests were performed on day 36, and animals were killed on day 40 for BrdU staining.

### RNA interference

Synapsin Ia/b shRNA(m) lentiviral particles, we named it LV-synapsin-shRNA-GFP, and its control shRNA lentiviral particles (LV) were purchased (Santa Cruz, CA. USA). LV-synapsin-shRNA is a pool of concentrated, transduction-ready viral containing 3 target-specific constructs that encode 19–25 nt (plus hairpin) shRNA designed to knock down gene expression. Each viral contains 200 μl frozen stock containing 1.0 × 10^6^ infectious units of virus (IFU) in Dulbecco’s Modified Eagle’s Medium with 25 mM HEPES pH 7.3.

### Statistical analysis

Data are presented as means ± SEM. The significance of differences was determined using one-way ANOVAs followed by Scheffe’s post hoc test. Differences were considered significant when p < 0.05.

## Additional Information

**How to cite this article**: Zhang, J. *et al*. CREB-mediated synaptogenesis and neurogenesis is crucial for the role of 5-HT1a receptors in modulating anxiety behaviors. *Sci. Rep.* **6**, 29551; doi: 10.1038/srep29551 (2016).
