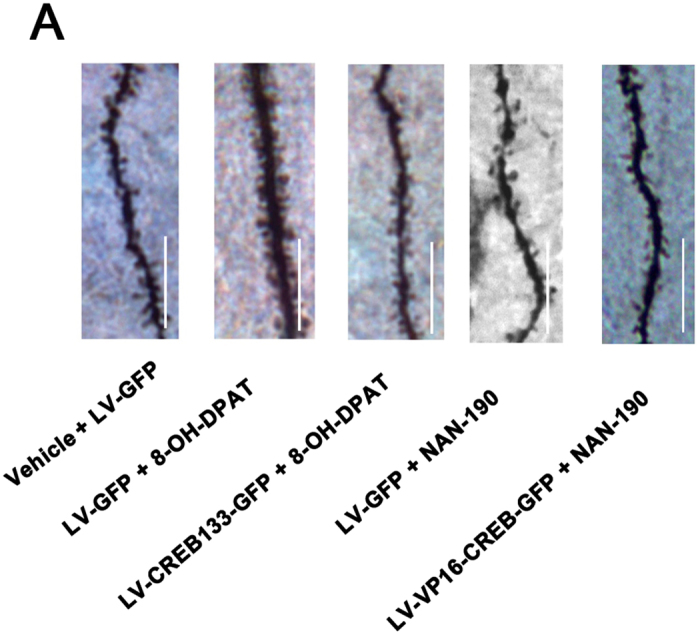# Corrigendum: CREB-mediated synaptogenesis and neurogenesis is crucial for the role of 5-HT1a receptors in modulating anxiety behaviors

**DOI:** 10.1038/srep43405

**Published:** 2017-02-24

**Authors:** Jing Zhang, Cheng-Yun Cai, Hai-Yin Wu, Li-Juan Zhu, Chun-Xia Luo, Dong-Ya Zhu

Scientific Reports 6: Article number: 2955110.1038/srep29551; published online: 07 12 2016; updated: 02 24 2017

This Article contains an error in Figure 4A, where the LV-VP16-CREB-GFP + NAN-190 representative image is incorrect. The Figure legend is correct. The correct Figure 4A appears below as Figure 1.

## Figures and Tables

**Figure 1:**